# The Impact of Sample Storage on Blood Methylation: Towards Assessing Myelin Gene Methylation as a Biomarker for Progressive Multiple Sclerosis

**DOI:** 10.3390/ijms25063468

**Published:** 2024-03-19

**Authors:** Assia Tiane, Veerle Somers, Niels Hellings, Daniel L. A. van den Hove, Tim Vanmierlo

**Affiliations:** 1 Department of Neuroscience, Biomedical Research Institute, Faculty of Medicine and Life Sciences, Hasselt University, 3500 Hasselt, Belgium; 2 Department Psychiatry and Neuropsychology, School for Mental Health and Neuroscience, Maastricht University, 6229 Maastricht, The Netherlands; d.vandenhove@maastrichtuniversity.nl; 3 University MS Center (UMSC), 3500 Hasselt, Belgium; veerle.somers@uhasselt.be (V.S.); niels.hellings@uhasselt.be (N.H.); 4 Department of Immunology and Infection, Biomedical Research Institute, Faculty of Medicine and Life Sciences, Hasselt University, 3500 Hasselt, Belgium; 5 Department of Psychiatry, Psychosomatics and Psychotherapy, University of Wuerzburg, 97080 Wuerzburg, Germany

**Keywords:** biomarker, DNA methylation, (re)myelination, multiple sclerosis, epigenetics

## Abstract

One of the major challenges in multiple sclerosis (MS) is to accurately monitor and quantify disability over time. Thus, there is a pressing need to identify new biomarkers for disease progression. Peripheral blood DNA methylation has been demonstrated to be an easily accessible and quantifiable marker in many neurodegenerative diseases. In this study, we aimed to investigate whether methylation patterns that were previously determined in chronic inactive white matter lesions of patients with progressive MS are also reflected in the blood, and whether the latter can serve as a biomarker for disease progression in MS. While our initial analysis revealed differences in the blood methylation state of important myelin-related genes between patients with progressive MS and controls, these findings could not be validated in other independent patient cohorts. Subsequent investigation suggests that sample storage can selectively influence DNA methylation patterns, potentially hindering accurate epigenetic analysis. Therefore, sample storage time should be taken into consideration during the initial sample selection stage in biomarker studies.

## 1. Introduction

Multiple sclerosis (MS) affects around 2.5 million people globally, causing a high healthcare burden [1]. Around 85% of all patients with MS are initially diagnosed with a relapsing–remitting disease course (RRMS), of which more than 50% will end up developing progressive MS within a period of 10–15 years, independent of treatment [2]. Progressive MS is mainly characterized by the accumulation of chronically demyelinated lesions, as a consequence of failed endogenous remyelination. Sustained axonal damage within these lesions eventually leads to neurodegeneration, as reflected by the progressive clinical disability of these patients with MS [3,4,5,6].

One of the major challenges in MS is to identify new biomarkers that allow us to access the level of progression of the disease. Current diagnostics are based on a combination of magnetic resonance imaging (MRI), neurologic examinations (such as the Expanded Disability Status Scale, EDSS), and the patient’s clinical history, concomitant with several limitations [2,7]. Diagnosis of progressive MS requires documented progression of disability over a period of at least six months, with the consequence of a delayed diagnosis of up to three years in some cases [8]. Furthermore, the lack of specific and sensitive markers for disease activity and progression does not only impact clinical decision making, but also slows down the discovery and validation of new therapeutic agents, as current clinical trials mainly depend on traditional clinical imaging outcomes, such as brain atrophy [9,10]. Thus, there is an urgent need for easily accessible, quantifiable, and reliable biomarkers for disease progression, associated with remyelination impairment or recovery capacity. The discovery of such biomarkers may further provide new insights into the pathological mechanisms that underlie progressive MS, accelerate and facilitate clinical trials, and therefore lead to new therapies for progressive MS.

Epigenetic processes, in particular DNA methylation, play a crucial role in oligodendrocyte precursor cell (OPC) differentiation and remyelination [11,12,13]. DNA methylation has gained great interest over the past years in its application as a biofluid biomarker for neurodegenerative diseases [14,15,16,17]. To some extent, blood DNA methylation patterns reflect the brain methylome, either by the presence of cell-free DNA derived from the brain due to blood–brain barrier leakage, or by reflecting a systemic epigenetic imprint affecting the methylation state of both brain cells and peripheral blood mononuclear cells (PBMCs) [18,19]. Taken together, this provides an incentive to investigate blood-borne methylation profiles as accessible biomarkers for monitoring the progression and course of demyelinating diseases, which reflect the epigenetic imprinting in the central nervous system (CNS). We have previously shown that CNS lesions of patients with progressive MS have a distinct DNA methylation signature related to the regulation of myelin gene expression [20]. We therefore investigated whether the blood methylomic profile of myelin-related genes is systemically altered in progressive MS stages and can serve as a blood-borne biomarker for demyelination. Newly identified biomarkers could be used to closely monitor ongoing brain damage throughout the course of the disease and may serve as targets for the development of successful treatment regimens for progressive MS.

## 2. Results

**Figure 1:**
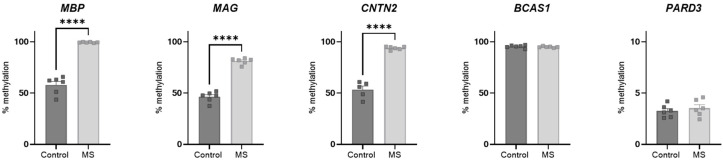
Myelin-related genes show a differentially methylated profile when comparing peripheral blood samples from non-neurological controls and patients with multiple sclerosis (MS). DNA isolated from peripheral blood samples from the same MS patients as the brain discovery (NBB) cohort was used for bisulfite pyrosequencing. Three out of five genes displayed significant differential methylation compared to controls. Data are represented as mean + SEM. Unpaired *t*-test, **** = *p* < 0.0001.

We have previously shown that genes related to myelination and oligodendrocyte differentiation are differentially methylated in chronically demyelinated lesions of patients with progressive MS [20]. As DNA methylation has gained interest in its application as a biofluid biomarker for neurodegenerative diseases, we hypothesized that the methylation signature of these genes could also be detected in peripheral blood samples of patients with progressive MS, reflecting ongoing brain pathology. We therefore obtained DNA from whole blood samples of the same patients as the discovery brain cohort, as well as age- and sex-matched non-neurological control samples (Table 1). DNA methylation of five of the top differentially methylated myelin-associated genes [myelin basic protein (*MBP)*, myelin-associated glycoprotein *(MAG)*, contactin 2 *(CNTN2)*, brain-enriched myelin-associated protein 1 *(BCAS1)*, partitioning defective 3 homolog *(PARD3*)] was assessed using bisulfite pyrosequencing [20]. Three (*MBP, MAG, CNTN2*) out of the five genes showed a significant difference in methylation pattern between the control and MS samples (Figure 1). Interestingly, the methylation profile of these genes followed the pattern observed in the CNS, suggesting systemic hypermethylation of these genes in patients with progressive MS. This suggests that the DNA methylation profile of these genes could be applied in a peripheral marker panel for assessing demyelination in the disease course of MS.

**Figure 2:**
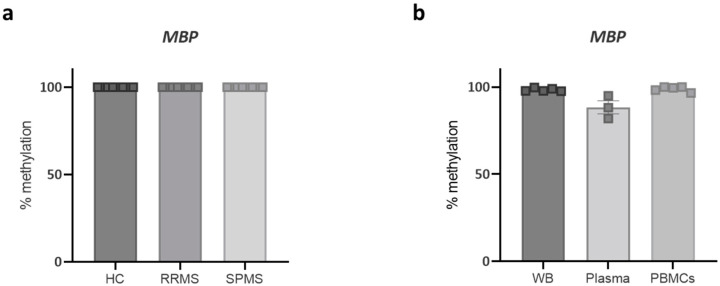
Lack of differential methylation of *MBP* in UBiLim blood cohorts. (**a**) DNA from plasma samples of healthy controls, relapsing–remitting (RRMS) patients, and secondary progressive (SPMS) patients does not exhibit any difference in *MBP* methylation. (**b**) DNA isolated from different blood fractions of healthy control samples shows an overall hypermethylated profile of *MBP* in all the blood fractions. Data are represented as mean ± SEM. HC = healthy control, WB = whole blood, PMBCs = peripheral blood mononuclear cells.

An ideal biomarker for progression in MS would be able to differentiate between the RRMS and SPMS stages. Therefore, in the next experiment, we made use of DNA isolated from plasma from healthy controls, patients with RRMS, and patients with SPMS, obtained from a different biobank, and evaluated the methylation profile of one of the myelin-related genes, *MBP*. To our surprise, we found no difference between the three groups. In contrast to our previous findings, healthy controls now also exhibited a hypermethylated profile (Figure 2a). Since we observed significant differences in *MBP* methylation in DNA isolated from whole blood samples in our discovery cohort, it is evident that the discrepancy could be attributed to the absence of PBMCs in the plasma samples of the new cohort. Therefore, in the next step, we isolated DNA from different fractions (whole blood, plasma, and PBMCs) from new healthy control donors. Interestingly, the *MBP* gene displayed hypermethylation in all three blood fractions (Figure 2b).

**Figure 3:**
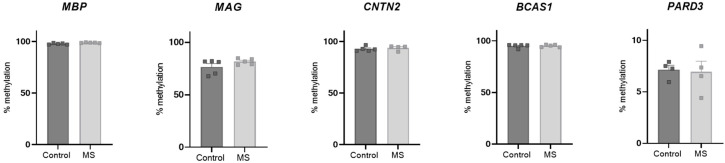
Samples with a shorter storage time do not show differences in methylation between controls and multiple sclerosis (MS) samples. DNA isolated from peripheral blood samples from a new cohort of patients with MS and non-neurological controls, with a maximum storage period of ten years, was used for bisulfite pyrosequencing. Data are represented as mean ± SEM.

The discrepancy in our data suggests that the original findings from the discovery cohort may have been biased by certain covariates. We therefore performed a correlation analysis between the methylation values of *MBP* and different covariates, such as age, sex, postmortem interval (PMI), and storage time (Table 2). Age, sex, and PMI did not show any correlation with *MBP* methylation. Interestingly, storage time, the only covariate that was not matched between the groups (control vs. MS) during the selection of the samples, exhibited a strong and significant negative correlation with the *MBP* methylation state (Table 2). These results suggest that a long storage time of the samples may lead to the loss of certain DNA methylation signatures. To investigate this, we obtained new samples from the NBB, with an inclusion criterion of a storage time of less than 10 years (Table 3). We performed bisulfite pyrosequencing on these samples for the five myelin-related genes. Interestingly, and in contrast to our previous findings, we observed no differences between the two groups for any of the measured genes (Figure 3). The hypomethylated profile of the control samples was not reproducible in the new sample cohort with a shorter storage time. The hypomethylated pattern observed in the control samples from the initial cohort was not replicated in the new sample set, which had been stored for a shorter duration. To rule out potential technical errors, we included control and MS samples from the original cohort in this analysis, consistently finding hypomethylation in that control sample (Supplementary Figure S1). Collectively, these data show that the DNA methylation profile of specific genetic regions can be strongly influenced by sample storage time.

## 3. Discussion

The original aim of the present study was to investigate whether the DNA methylation state of myelin-related genes could be applied as a blood-borne biomarker for disease progression in MS. While we initially observed significant differences in peripheral DNA methylation between patients with MS and non-neurological controls, we were not able to reproduce these findings in samples obtained from other cohorts. Notably, we observed a strong correlation between the degree of DNA methylation of these genes and the storage time of the samples. Our data suggest that the DNA methylation signature can be affected by long-term storage conditions, an important factor that should be considered in future studies.

The diagnosis of progressive MS is still considered a significant challenge, as there are currently no accessible quantifiable markers available [7,21]. The current examination of the transition from RRMS to SPMS is mainly based on retrospective analysis of clinical parameters, which means that the transition to progressive MS can remain unnoticed with a delay of up to three years [21,22]. Upon the recent approval of disease-modifying drugs for SPMS, such as siponimod, an increasing need for timely and accurate observation from the RRMS to the SPMS stage has developed [23]. As for the development of new drugs that modulate the disease progression, biomarkers for remyelination impairment can be applied in drug screening phases, as well as in human clinical trials. Such markers could provide accurate and valid indications of the effect of a treatment on patients, thereby enabling and accelerating smaller clinical trials [9]. There is a great effort within the MS research domain to discover new accessible and quantifiable markers for disease progression. For example, the integration of MRI data with proteomic data from the cerebrospinal fluid (CSF) of patients was able to distinguish between RRMS and SPMS stages [24]. Similarly, the combination of MRI data with cognitive performance accurately discriminated patients with RRMS from patients with SPMS [25]. A new PET tracer ([18F]3F4AP) could effectively detect myelin loss in primates and is currently being tested in a clinical study with healthy volunteers and patients with MS, with the final aim to apply this tracer as a new in vivo imaging tool for demyelination [26,27]. At the molecular level, diagnostic markers like serum neurofilament light chain (sNFL) levels have gained significant attention for their potential use as markers for disease progression in MS. Despite its prognostic value in MS, sNFL level predominantly reflects neuroaxonal injury, a phenomenon observed in various neurodegenerative diseases like Alzheimer’s disease and frontotemporal dementia, making it less specific for (progressive) MS [28]. To date, there are no easily accessible, reliable, and specific markers that can define the progressive phase of MS or anticipate the conversion to SPMS.

DNA methylation has gained great interest in its application as a biomarker for many neurodegenerative or neuropsychiatric diseases. In Parkinson’s disease, Alzheimer’s disease, and epilepsy, for instance, numerous DNA methylation signatures in peripheral blood samples have been shown to reflect methylation differences within the brain [16,29,30]. We therefore wondered whether the epigenetic differences we previously observed in MS brain samples could be detected in peripheral blood samples and used as markers for progression in MS. We have previously identified important genes regarding myelination and oligodendrocyte differentiation, which were hypermethylated in chronically demyelinated lesions of patients with progressive MS [20]. In the present study, we took a hypothesis-driven approach to investigate the methylation state of these genes in whole blood samples, isolated from the same patients as the brain discovery cohort. Interestingly, three genes (*MBP*, *MAG*, *CNTN2*) initially seemed to be significantly hypermethylated in MS blood samples compared to the non-neurological control samples. Methylation of the CpG-rich promotor region is generally associated with gene silencing due to the inhibition of transcription factor binding. In the CNS, epigenetic silencing of these important myelin-related genes could be one of the underlying factors contributing to the inhibition of OPC differentiation into myelin-forming oligodendrocytes. Similarly, the myelin oligodendrocyte glycoprotein (MOG) gene, another important myelin gene, has previously been described to be demethylated in serum from patients with MS with an active and symptomatic disease course, probably reflecting oligodendrocyte cell loss during these stages of the disease [19].

Our main goal was to define a specific marker for disease progression in MS, which would therefore distinguish patients with SPMS from patients with RRMS. We isolated DNA from the plasma of a new cohort of healthy control subjects and age- and sex-matched patients with RRMS and SPMS. Unexpectedly, we observed no differences in methylation between the three groups, as all samples showed a hypermethylated profile of *MBP* in this cohort. A possible explanation for this discrepancy in our data is that in the first cohort, we made use of DNA isolated from whole blood samples, including both cell-free DNA and DNA from PBMCs, whereas in the cohort with the different disease stages, we only looked at cell-free DNA isolated from plasma samples. The absence of PBMCs could thus be a potential factor influencing the reproducibility of our data. To confirm this, we isolated DNA from different blood fractions (whole blood, plasma, PBMCs) from healthy control donors. Interestingly, all the fractions showed the same hypermethylated state of *MBP* within the control samples, confirming that the observations from the first cohort were not reproducible. Nonetheless, a slight drawback in this context is that the blood–brain barrier (BBB) remains predominantly intact in healthy individuals, while patients with MS exhibit a leaky BBB, leading to the escape of brain-derived cell-free DNA. Consequently, analyzing solely healthy control blood fractions might have resulted in our overlooking this signal.

During the selection procedure of the samples of the first cohort, we matched the samples based on age, sex, and PMI. Notably, correlation analysis between the degree of *MBP* methylation and sample storage time, a variable that initially was not used as a selection criterion, showed a strong and significant correlation with the sample storage time. Indeed, when we included storage time (less than 10 years at 4 °C) as an inclusion criterion during the sample selection of a new set of DNA samples from whole blood, we no longer observed differences when comparing the controls and patients with progressive MS. These results suggest that a long storage time of the samples can result in a selective loss of the DNA methylation signature at specific genetic regions. Previous studies have already investigated the stability of DNA methylation marks after long-term storage [31,32]. Interestingly, no global changes in DNA methylation were observed after 20 years of storage of DNA samples at 4 °C in another study [32]. We did observe a loss of methylation in three out of the five measured genes in the control DNA samples stored for more than 20 years at 4 °C. As this loss of methylation was not observed for all genes, this selective loss of methylation could be missed during the screening of global methylation changes of archived samples, as previously conducted by others [32]. Moreover, different storage conditions between different institutions and agencies may also play an important role in this respect. Evidently, the longer samples are being stored, the higher the likelihood that incidents, e.g., related to temporary changes in temperature, will occur. Interestingly, matching brain samples, which were stored at −80 °C for the same time period, did not show a loss of methylation of the measured genes. Altogether, our data suggest that the DNA methylation signature in blood can be affected by long-term storage, an important factor that had been previously overlooked yet should be considered in future studies.

Unfortunately, in the present study, we were not able to discover new biomarkers for progression in MS based on the DNA methylation of a subset of myelin-related genes. Our targeted approach, based on genes that displayed differential methylation in chronically demyelinated lesions compared to the surrounding non-affected white matter, proved unsuccessful. Therefore, it would be of great interest to subject DNA from peripheral blood samples, whether whole blood or plasma, from both patients with progressive MS and control individuals to genome-wide methylation analysis. Data analysis comparing cases versus controls in both brain tissue and peripheral blood could then reveal potential differentially methylated genes that overlap between the brain and periphery. These genes could be further investigated for their role as a biomarker for disease progression, reflecting the ongoing CNS pathology. To this end, it would be essential to expand the sample size and incorporate diverse cohorts with varied demographic and clinical characteristics, which could provide more comprehensive insights into the application of DNA methylation patterns as a biomarker for progressive multiple sclerosis.

## 4. Materials and Methods

### 4.1. Study Cohorts and Ethical Approval

DNA isolated from whole blood from two cohorts of patients with MS and non-neurological controls was provided by the Netherlands Brain Bank (NBB). Demographic characteristics of both cohorts are described in Table 1 and Table 2, respectively. Plasma samples of healthy controls, patients with relapsing–remitting MS (RRMS), and patients with secondary progressive MS (SPMS), as well as blood fractions (whole blood, plasma, PBMCs), were provided by the UBiLim biobank. All experiments were conducted after approval by the ethical committee of Hasselt University, and patient anonymity was assured by handling the tissue samples in a coded fashion.

### 4.2. Pyrosequencing

Genomic DNA was extracted from PBMCs, plasma, or whole blood and bisulfite-converted using the Zymo Research EZ DNA Methylation-Direct Kit (BaseClear Lab Products). PCR primers were designed using the PyroMark Assay Design 2.0 software (Qiagen, Antwerp, Belgium, Supplementary Table S1). The assays always included an internal bisulfite control and were tested for their sensitivity using the EpiTect PCR Control DNA Set (Qiagen, Antwerp, Belgium). Product amplification was performed using the following reaction mixture: 1× Buffer with 20 mM MgCl_2_ (Roche, Vilvoorde, Belgium), 10 mM dNTP mix (Roche, Vilvoorde, Belgium), 5 μM forward and reverse primers (Metabion AG, Planegg, Germany), 1U FastStart Taq DNA Polymerase (Roche, Vilvoorde, Belgium), bisulfite-converted DNA, and nuclease-free water to a total volume of 25 μL. PCR cycling was performed as follows: initial denaturation for 5 min at 95 °C; 50 cycles of 30 s at 95 °C, 30 s at the appropriate annealing temperature, and 1 min at 72 °C; and final extension for 7 min at 72 °C. PCR amplicons were sequenced using the PyroMark Q48 instrument (Qiagen, Antwerp, Belgium) with the PyroMark Q48 Advanced CpG Reagents (Qiagen, Antwerp, Belgium), according to the manufacturer’s protocol, and quantified with the PyroMark Q48 Autoprep software version 4.3.3 (Qiagen, Antwerp, Belgium).

### 4.3. Statistical Analysis

Statistical analysis was performed using GraphPad Prism 9.0.0 software (GraphPad software Inc., La Jolla, CA, USA). Differences between group means were determined using an unpaired *t*-test. Correlation analyses were performed using Spearman’s correlation tests. All data are depicted as mean ± SEM; * = *p* ≤ 0.05, ** = *p* < 0.01, *** = *p* < 0.001, **** = *p* < 0.0001.

## Figures and Tables

**Table 1:** Demographic details of the first cohort of peripheral blood DNA samples obtained from the Netherlands Brain Bank (NBB).

Group	Sex (F/M)	Age (Mean ± SD)	PMI (Mean Minutes ± SD)	Storage Time (Mean Years ± SD)
MS	2/4	66.50 ± 9.69	572.5 ± 81.16	9.33 ± 1.63
Controls	3/3	64.33 ± 6.09	605.0 ± 284.7	24.67 ± 2.25

PMI: postmortem interval.

**Table 2:** Storage time correlates significantly with the methylation state of *MBP*. Pearson’s correlation analysis was performed between *MBP* methylation and different covariates, such as age, sex, PMI, and storage time. PMI = postmortem interval.

Covariate	Pearson r	*p* Value
Age	0.1559	0.6286
Sex	−0.2880	0.3641
PMI	−0.0065	0.9839
Storage time	−0.9129	<0.0001

**Table 3:** Demographic details of the second cohort of peripheral blood DNA samples obtained from the Netherlands Brain Bank.

Group	Sex (F/M)	Age (Mean ± SD)	PMI (Mean Minutes ± SD)	Storage Time (Mean Years ± SD)
MS	3/2	68.00 ± 18.61	524.0 ± 92.29	9.20 ± 1.30
Controls	3/2	69.20 ± 17.30	440.0 ± 37.58	9.00 ± 1.41

## Data Availability

Data is contained within the article and Supplementary Materials.

## Supplementary Materials

The supporting information can be downloaded at: https://www.mdpi.com/article/10.3390/ijms25063468/s1.
