# Supplementary material for: The Impact of Sample Storage on Blood Methylation: Towards Assessing Myelin Gene Methylation as a Biomarker for Progressive Multiple Sclerosis
Source: Int J Mol Sci. 2024 Mar 19;25(6):3468. doi: 10.3390/ijms25063468 (PMC10970687; doi:10.3390/ijms25063468)
Supplement: Supplementary file 1 [file ijms-25-03468-s001.zip › ijms-2851505-supplementary.pdf]

Supplementary information

Supplementary Table S1: Pyrosequencing primer list

Target gene	Forward primer (5'-3')	Reverse primer (5'-3')	Sequencing primer (5'-3')
MBP	GTTTGGTAGGATGTTTATTTAGTTGA	TCTATAACCCCATCACATCCAAACTCTC	GGATGTTTATTTAGTTGATTTAGG
MAG	AGGGTGTATAGGGATGGAAGAT	AAAAAAAACACAAAAAAAACCCTTATCAC	GGAAAGAGTTAGGAGAATTTA
CNTN2	GAGGGGGGTGAGATAATAGT	CCTACCAACTCTAAAATTCTAAATACTCA	TGAGATAATAGTGATAGTTTGA
BCAS1	GGAGTATATAGTTGAGGGGGTTGATAG	CTCAAAAACTTAAACTCTAACCCTAAATT	GGAAGTATAGTAGTTTGTTTATAAT
PARD3	AGGGAGAGGGTAGGGTAGAAA	CCCCTTCCCCTTTCTTTATC	AGAAATTTAGTAGAGTAAGTTGTAG

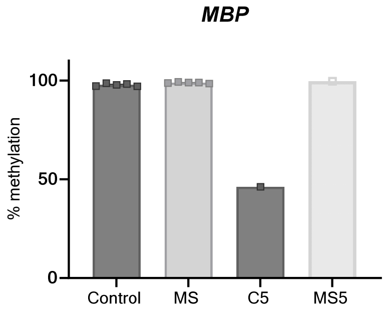

Supplementary Figure S1: To exclude the possibility of technical artifacts, we always included a control (C5) and an MS (MS5) sample from cohort 1 in the other experiments, consistently observing hypomethylation in the control sample.
